# Exploring molecular mechanisms of intra-articular changes in osteonecrosis of femoral head using DIA proteomics and bioinformatics

**DOI:** 10.1186/s13018-023-04464-3

**Published:** 2024-01-03

**Authors:** Gang Zhao, Yujie Liu, Yongjun Zheng, Mingyang An, Jia Zhang, Jing Zhang, Zhongli Li, Li Chunbao

**Affiliations:** 1 Department of Orthopedics, the No.4 Medical Centre, Chinese PLA General Hospital, Beijing, 100048 China; 2 Department of Orthopaedics, Chinese PLA 984 Hospital, Beijing, 100029 China; 3 Medical school of Chinese PLA, Beijing, 100853 China

**Keywords:** Osteonecrosis of the femoral head, DIA proteomics, Bioinformatics analysis, Differentially expressed proteins, Key regulatory proteins

## Abstract

**Purpose:**

This study is aimed to delve into the crucial proteins associated with hormonal osteonecrosis of the femoral head (ONFH) and its intra-articular lesions through data-independent acquisition (DIA) proteomics and bioinformatics analysis.

**Methods:**

We randomly selected samples from eligible ONFH patients and collected samples from the necrotic area of the femoral head and load-bearing cartilage. The control group comprised specimens from the same location in patients with femoral neck fractures. With DIA proteomics, we quantitatively and qualitatively tested both groups and analyzed the differentially expressed proteins (DEPs) between groups. Additionally, we enriched the analysis of DEP functions using gene ontology terms and Kyoto Encyclopedia of Genes and Genomes pathways and verified the key proteins in ONFH through Western blot.

**Results:**

Proteomics experiment uncovered 937 common DEPs (422 upregulated and 515 downregulated) between the two groups. These DEPs mainly participate in biological processes such as hidden attributes, catalytic activity, molecular function regulators, and structural molecule activity, and in pathways such as starch and sucrose metabolism, ECM–receptor interaction, PI3K-Akt signaling, complement and coagulation cascades, IL-17 signaling, phagosome, transcriptional misregulation in cancers, and focal adhesion. Through protein–protein interaction network target gene analysis and Western blot validation, we identified C3, MMP9, APOE, MPO, LCN2, ELANE, HPX, LTF, and THBS1 as key proteins in ONFH.

**Conclusions:**

With DIA proteomics and bioinformatics analysis, this study reveals the molecular mechanisms of intra-articular lesions in ONFH. A correlation in the necrotic area and load-bearing cartilage of ONFH at ARCO stages IIIB-IV as well as potential key regulatory proteins was identified. These findings will help more deeply understand the pathogenesis of ONFH and may provide important clues for seeking more effective treatment strategies.

## Introduction

Osteonecrosis of the femoral head (ONFH) is a severe global disease that has substantially impacted the health and quality of life of people for long time. Epidemiological data indicate over two million patients worldwide are battling this disease, and hundreds of thousands of new cases are diagnosed in China each year [1]. ONFH can cause joint pain and potentially lead to femoral head collapse or hip joint destruction, greatly disrupting the daily activities and quality of life of patients [2, 3].

ONFH is a complex polygenic disease associated with genetic factors, environmental influences, and their interactions. The recognized pathogenic mechanisms of non-traumatic ONFH include impaired blood flow, intraosseous compartment syndrome, lipid metabolism disorder, increase in intraosseous pressure, local intravascular coagulation, and osteocyte apoptosis hypothesis [4]. Therefore, investigation into the molecular mechanism of ONFH, identification of reliable biomarkers, and discovering of therapeutic targets are crucial [5]. Only through these means can researchers develop targeted strategies to prevent and treat ONFH.

In the past decade, many studies have focused on identifying susceptible sites of ONFH [6–8]. With the continuous development of high-throughput sequencing technology, extensive research on the gene expression products of ONFH has identified thousands of differentially expressed genes, many of which can serve as biomarkers for early diagnosis and molecular targeted treatment [9, 10]. We believe that proteins, as the final products of gene expressions, directly participate in and regulate all bioactivities. Proteomics, a technique that deeply dissects protein expressions and functions, can provide a new perspective. By studying proteins, we can further understand life processes, including disease onset and progression. Detection of specific proteins allows to diagnose diseases, evaluate disease progression, and quickly and accurately monitor treatment effects.

Our previous data-independent acquisition (DIA) quantitative proteomics analysis [11] revealed the upregulated or downregulated differential factors in the necrotic area of the femoral head and cartilage of steroid-induced ONFH patients compared to normal femoral heads. By screening candidate proteins and conducting enrichment and pathway analysis, we constructed protein–protein interaction (PPI) networks. These differences were comprehensively interpreted and understood using bioinformatics tools, such as gene ontology (GO) and Kyoto Encyclopedia of Genes and Genomes (KEGG) analyses. In this study, we hope to reveal the potential pathogenesis of ONFH and provide potential for discovering new therapeutic targets, thus offering fresh insights and strategies for the prevention and treatment of ONFH.

## Subjects and methods

### Inclusion and exclusion criteria

Inclusion criteria were: (1) diagnosis with ARCO stages III or IV ONFH, experiencing severe hip pain accompanied by limping; (2) willingness to undergo total hip arthroplasty; (3) age ≥ 18 years; (4) generally healthy without serious hereditary family diseases, and with essentially normal examination results; (5) meeting the diagnosis criteria of steroid-induced ONFH (i.e., using corticosteroids > 2 g prednisolone or equivalent within three months, developing disease within two years after corticosteroid treatment, having no other risk factors causing ONFH) [12].

Exclusion criteria were: (1) not meeting the above diagnostic, staging and inclusion criteria; (2) avascular necrosis of the femoral head due to hip dysplasia; (3) malignant tumors; (4) joint infection; (5) traumatic ONFH; (6) previously undergoing hip surgery; (7) femoral head samples that cannot provide sufficient protein concentration for detection due to various reasons.

### Subjects

We selected steroid-induced ONFH patients who were first diagnosed and treated at our hospital between August and September 2022. The control group consisted of patients treated at our hospital during the same period who underwent femoral head replacement surgery due to femoral neck fractures. All procedures and operations complied with the regulations of the Ethics Committee in our hospital.

## Methodology

### Collection of femoral head samples

All surgeries were performed in collaboration with the author's team. Necrotic femoral heads that met the inclusion and exclusion criteria were collected during total hip arthroplasty, placed in cryopreservation boxes, and immediately transferred into a thermal bucket filled with liquid nitrogen. The samples were then promptly moved to the clinical biosample repository of our hospital and stored at − 80 °C. All procedural actions were duly recorded.

Control samples were sourced from patients immediately undergoing total hip replacement following femoral neck fractures. Any risk factor or condition related to femoral head necrosis was excluded.

### Experimental procedures

#### Sample preparation

The femoral head samples were processed via protein extraction, denaturation, reduction alkylation, enzymolysis, and peptide desalting. We collected tissues from the necrotic area and the adjacent normal area in the femoral head necrosis samples. In the control group, the corresponding cancellous bone was taken from the femoral head. Cartilage from the weight-bearing area of the femoral heads was also collected from both groups.

For sample pre-processing, proteins were extracted, denatured, reduced, alkylated, and digested into peptides, which were then desalted. An iST sample pre-processing kit (PreOmics, Germany) was used to prepare tissue samples, which were ground with liquid nitrogen. Then, an appropriate amount of a sample was taken, mixed with 50 µl of a lysis buffer, and heated at 95 °C and 1000 rpm for 10 min. After cooling to room temperature, a trypsin digestion buffer was added, and the sample was incubated at 37 ℃ and 500 rpm for 2 h. The enzyme reaction was terminated by adding a stop buffer. Peptides were desalted using the iST cartridge included in the kit and eluted twice with 100 µl of an elution buffer each time. The eluted peptides were dried under vacuum and stored at – 80 ℃.

### Spectral database establishment

#### High pH reversed-phase separation

All peptide mixtures were re-dissolved in buffer A (20 mM ammonium formate solution, adjusted to pH 10.0 with ammonia) and separated using a reverse-phase column (XBridge C18 column, 4.6 mm × 250 mm, 5 μm, Waters Corporation, MA, USA) connected to an ultimate 3000 system (ThermoFisher scientific, MA, USA). The separation involved a linear gradient from 5 to 45% B over 40 min (B: 80% ACN added with 20 mM ammonium formate, adjusted to pH 10.0 with ammonia). The column was equilibrated under initial conditions for 15 min with a flow rate at 1 mL/min at 30 ℃. Six fractions were collected, which were then dried in a vacuum concentrator for subsequent use.

### Low pH nano-HPLC–MS/MS (DDA qualitative library building)

The desalted peptides were freeze-dried, re-dissolved in solvent A (0.1% formic acid solution) and analyzed by LC–MS/MS equipped with an online nanospray ion source. The entire system integrated an Orbitrap Lumos mass spectrometer connected to an EASY-nLC 1200 system (Thermo Fisher Scientific). Totally 3 μL of a sample was loaded onto an analytical column (Acclaim PepMap C18, 75 μm × 25 cm) and separated using a 120-min gradient: 5–35% B (B: 0.1% formic acid in ACN solution).

The mass spectrometer was run in the data-dependent acquisition mode and automatically switched between MS and MS/MS acquisitions. Spectrometric parameters were set as follows: (1) MS: scan range (m/z): 350–1500; resolution: 120,000; AGC target: 4e5; maximum injection time: 50 ms; dynamic exclusion duration: 30 s; (2) HCD-MS/MS: resolution: 15,000; AGC target: 5e4; maximum injection time: 35 ms; collision energy: 32.

### Database search

Raw data were merged and searched through Spectronaut X (Biognosys AG) using databases like Uniprot or provided databases. A contamination sequence library was also concurrently searched to identify possible contamination in the samples. Trypsin digestion was set. The search parameters included a fixed modification: Carbamidomethyl (C), and variable modifications: methionine oxidation. False discovery rates (FDRs) were set at 1% for both parent ions and peptides.

### DIA data acquisition

Each sample was suspended in 30 μL of solvent A (0.1% formic acid solution). Then, 9 μL of the resulting suspension was mixed with 1 μL of 10 × iRT peptides, separated using nano-LC and analyzed by online electrospray tandem mass spectrometry (MS/MS). The entire system integrated the mass spectrometer connected to the EASY-nLC 1200 system. Typically, 3 μL of a sample was loaded onto the analytical column and separated using a 120-min gradient from 5 to 35% solution B (B: 0.1% formic acid in ACN solution).

### Protein qualitative and quantitative analysis

Proteins extracted from the samples were enzymatically digested. Prior to mass spectrometry, the quality control reagent iRT Kit from Biognosys was added to each sample, which allowed for calibration based on the retention time (RT) of the peptides in the chromatogram. The raw MS data were quality-controlled using QuiC (Biognosys) to assess whether the QC indices were similar across all samples. If yes, the detection is well reproducible. Then, a library was built on Pulsar from the data obtained in the DDA acquisition mode, and DIA data were analyzed against the DDA reference database for protein identification [13]. If a protein was detected in at least one sample, the qualitative result of this protein and its quantitative results across all samples were reported.

### Protein annotation

GO is an internationally standardized gene function classification system that offers a dynamically updated standard vocabulary (Controlled Vocabulary) to comprehensively describe the attributes of genes and their products in biological organisms. GO encompasses three ontologies: Molecular Function, Cellular Component, and Biological Process. Pathway-based analysis further helps understand the coordinated functions of distinct proteins in executing their biological behaviors. Thus, we utilized KEGG to annotate the identified proteins at the level of biological pathways. KEGG integrates genomic, chemical and systemic functional information, and accumulates abundant annotations of biological pathways within organisms, which help identify major biochemical metabolic pathways and signal transduction pathways that involve proteins. Cluster of Orthologous Groups of Proteins (COG) is a database that classifies proteins into orthologous categories. Proteins that constitute each COG are assumed to originate from a common ancestral protein, i.e., orthologs and paralogs.

### PPI network analysis

In biological organisms, proteins do not exist independently. The functional expression of a protein often requires regulation and mediation by other proteins. The realization of this regulatory or mediating role first necessitates protein–protein binding or interactions [14]. Studying these interactions and the resulting networks is crucial for revealing the functions of proteins [15].

**Fig. 1:**
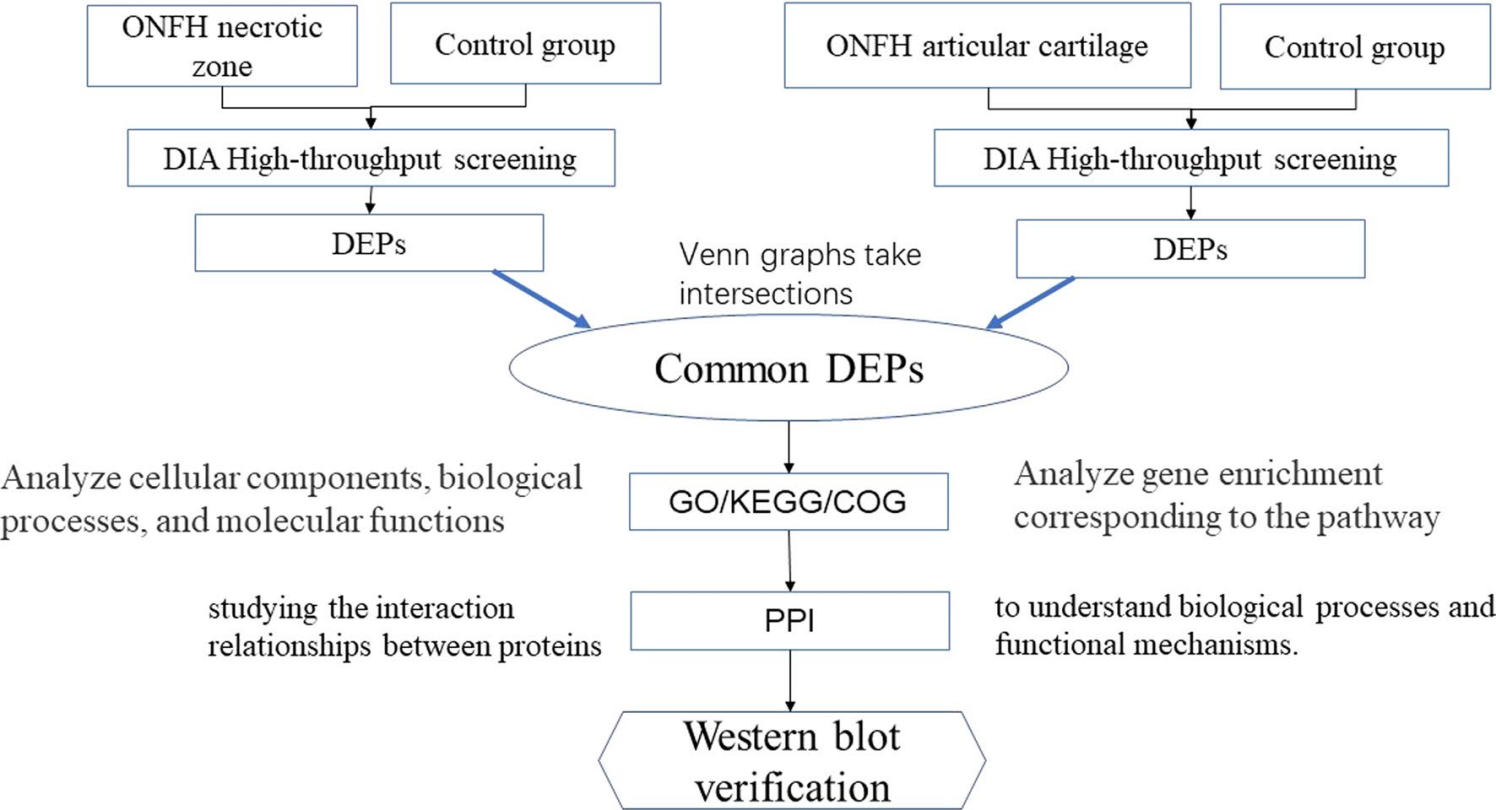
Flowchart

We mainly utilized interaction relationships from the protein interaction database STRING for differential protein interaction network analysis. For species included in the database, we extracted a set of differentially expressed proteins (DEPs) and constructed an interaction network diagram using Cytoscape [16]. The overall study design and analysis flowchart is shown in Fig. 1.

### Statistical analysis

Statistical analysis was done on SPSS 26.0. Results were presented as‾x ± s. Data following normal distribution were compared using Student's *t* test, while non-normally distributed data were analyzed using the Mann–Whitney *U* test. P < 0.05 indicates significance.

## Results

### Case sample collection

**Fig. 2:**
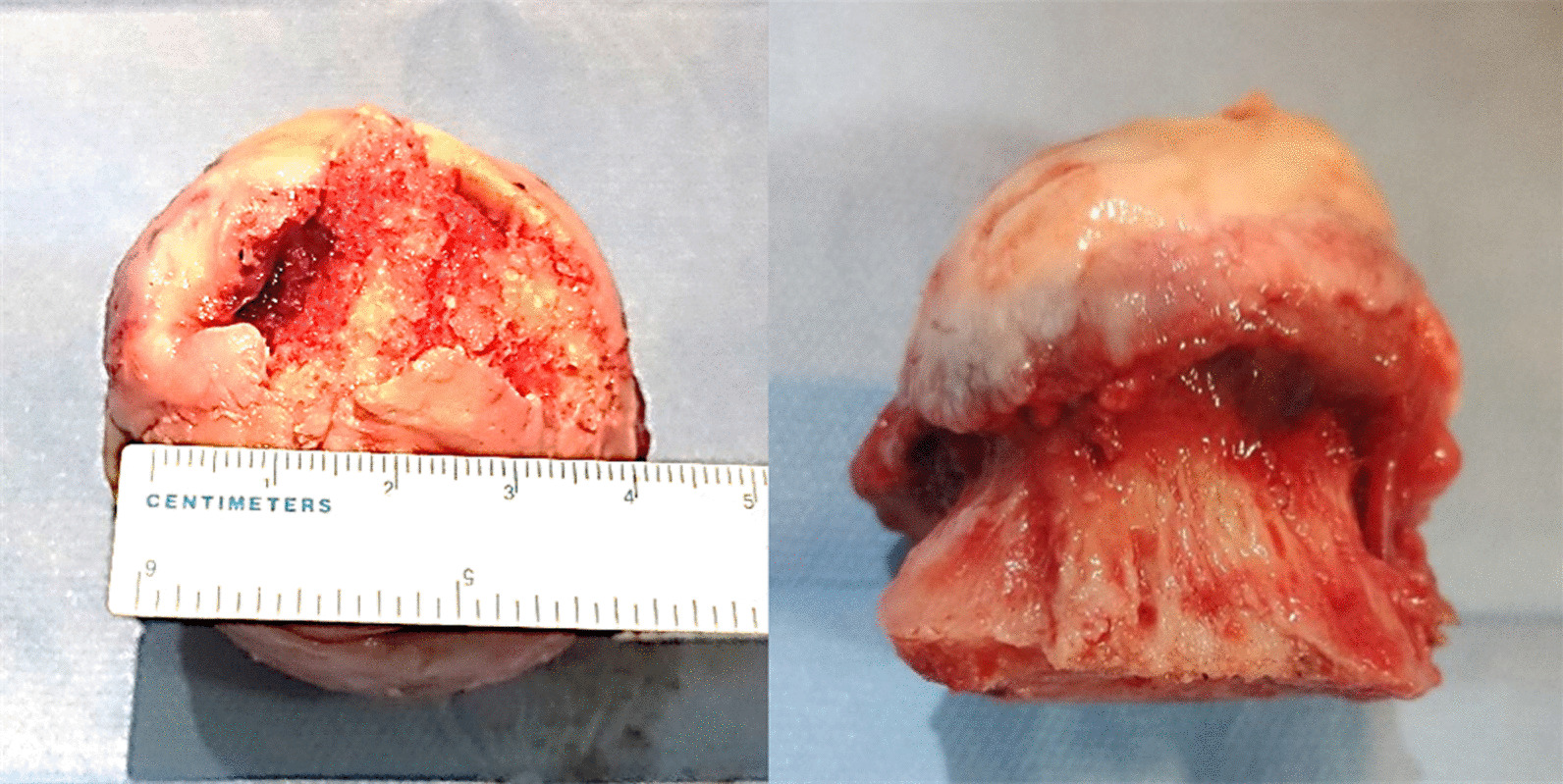
Collection of femoral head necrosis specimens with the collapse of the femoral head cartilage

**Fig. 3:**
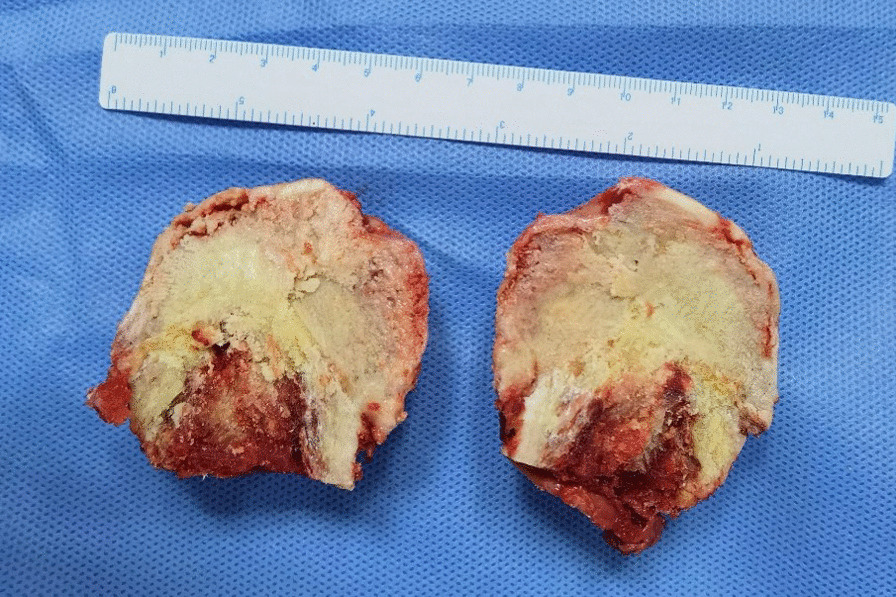
Cross section of the necrotic femoral head (red arrow: the area of collapse in the weight-bearing region)

**Fig. 4:**
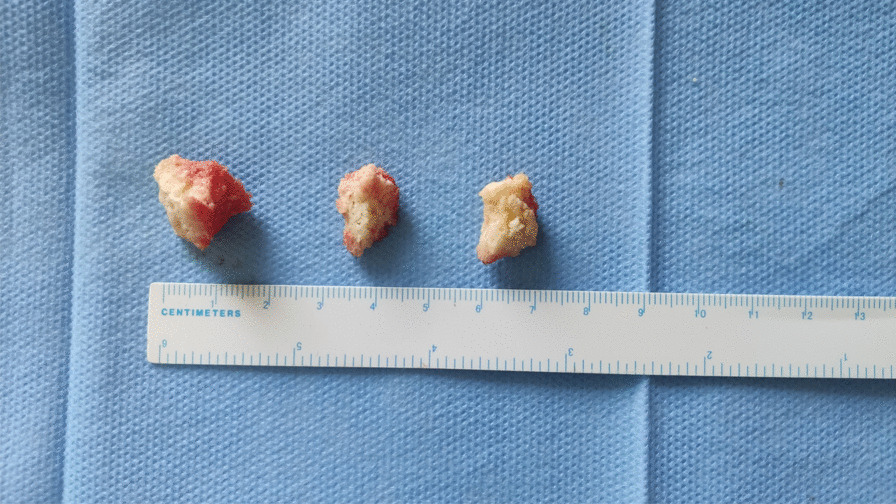
Bone tissues extracted from the necrotic area of the femoral head

This study included totally 13 cases of femoral head necrosis and 6 cases of femoral neck fractures diagnosed and treated between August and September 2022 (Fig. 2). Bone tissues from the necrotic area of the femoral head and cartilage tissues from the weight-bearing area of the femoral head were taken from the specimens. Moreover, cancellous bones and cartilages from corresponding locations were collected as the control group (Figs. 3 and 4).

### Protein qualitative and quantitative analysis

**Fig. 5:**
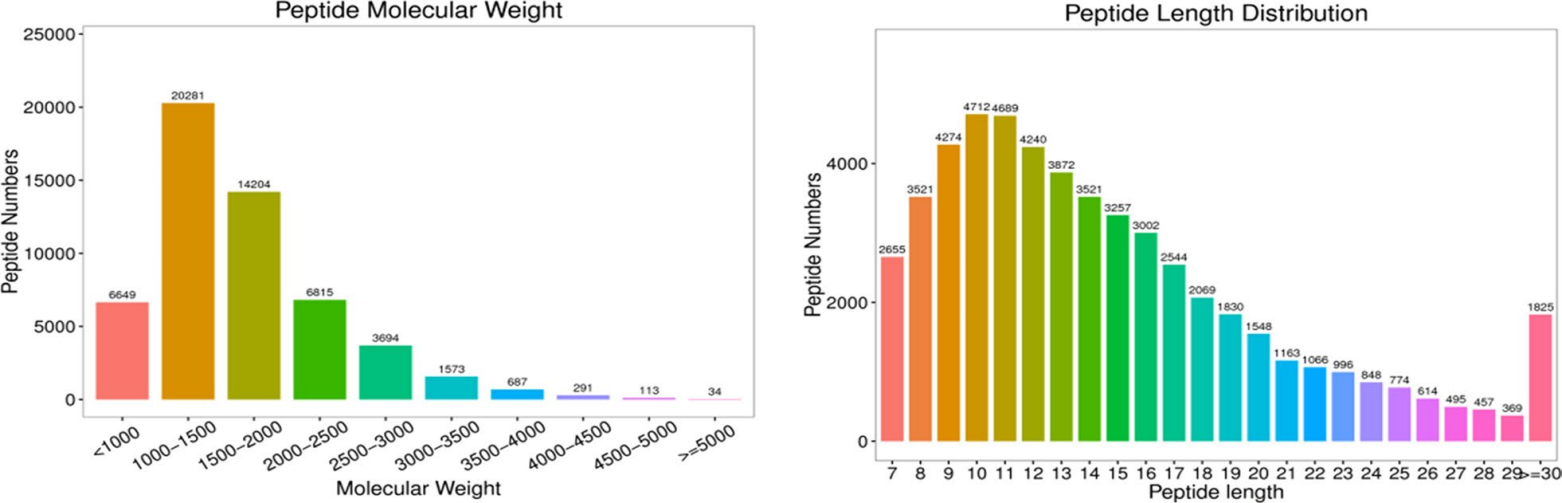
Sample molecular weight analysis

A total of 71,333 peptides, 5401 protein groups, and 5554 proteins were identified in the database built for machine detection. The molecular weights of most samples were between 1000 and 2500, and the peptide lengths were mostly distributed within 9–15 residues (Fig. 5).

### Sample relationship analysis

**Fig. 6:**
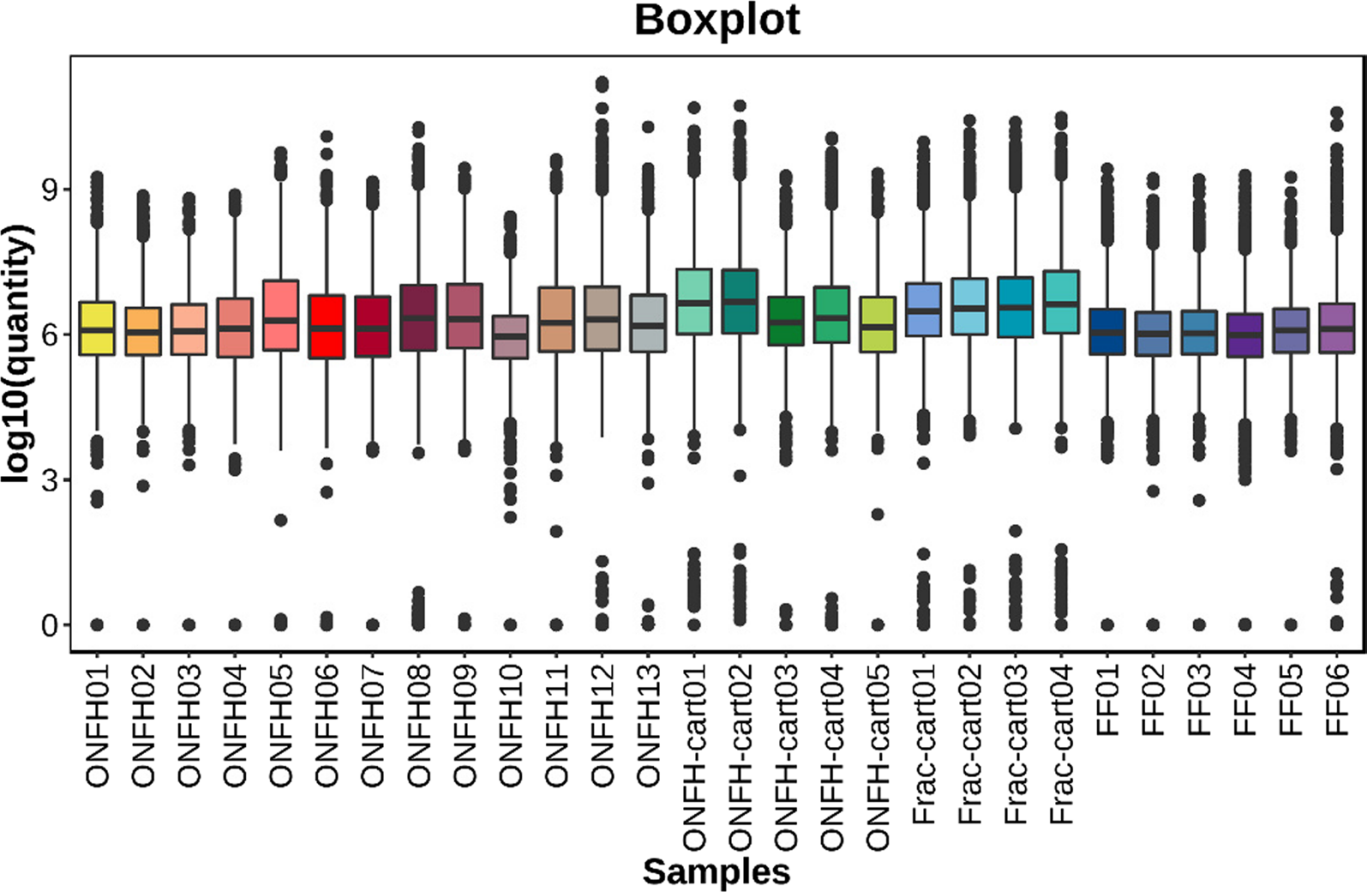
Box plot about the distribution of normalized peptide quantitative values. The signal strengths of most samples achieved roughly equivalent response intensities

The local normalization method in Pulsar was used to normalize overall sample peak intensities to avoid significant signal deviations in the samples with large background differences. This processing ensured the signal strengths of the samples achieved roughly equivalent response intensities, which made all samples comparable (Fig. 6).

**Fig. 7:**
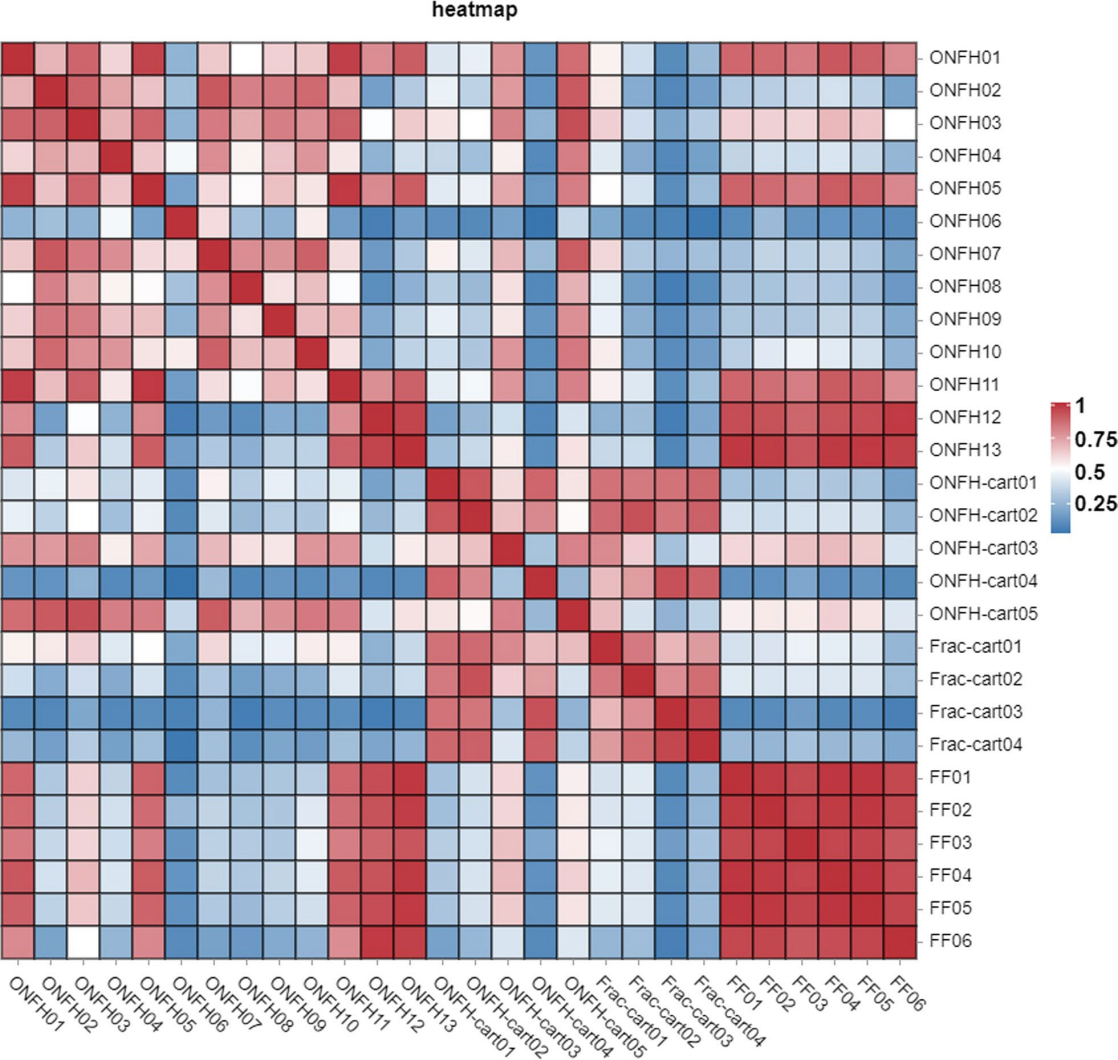
Heatmap

We can assess the quality of sample duplication within one group by comparing protein expressions between any two samples (Fig. 7).

**Fig. 8:**
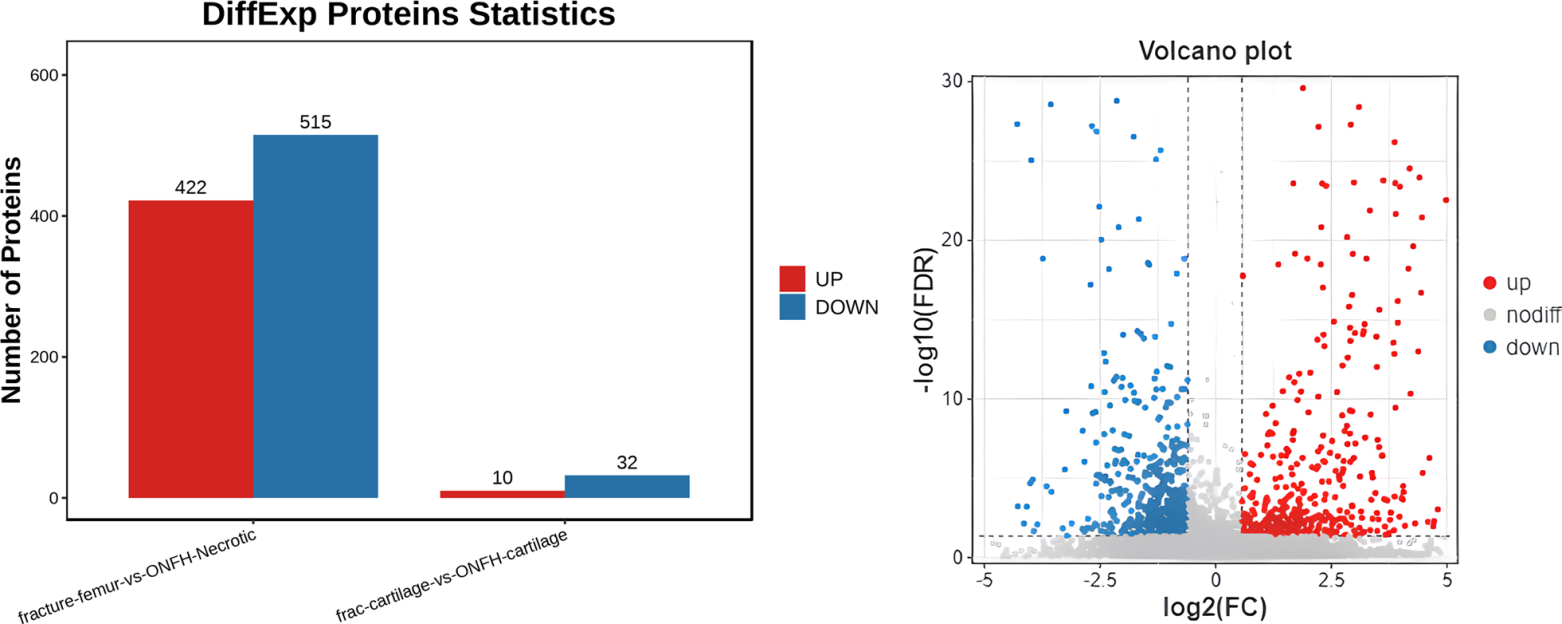
Volcano plots

Volcano plots showed upregulated and downregulated DEPs in red and blue, respectively (Fig. 8). All results were based on a corrected *P* < 0.05 and |log2FC|≥ 1 (FC: fold change) as the standards. Compared to the control group, 937 DEPs (422 upregulated and 515 downregulated DEPs) were found in ONFH. Only proteins with *P* ≤ 0.05 and |log2FC |≥ 1 were determined as DEPs.

### Protein annotation

The function and pathway enrichment of common upregulated and downregulated DEPs obtained from the intersection of three datasets from two chips was analyzed with the online database DAVID (https://david.ncifcrf.gov/tools.jsp), aiming to further explore the potential pathogenesis of ONFH.

**Fig. 9:**
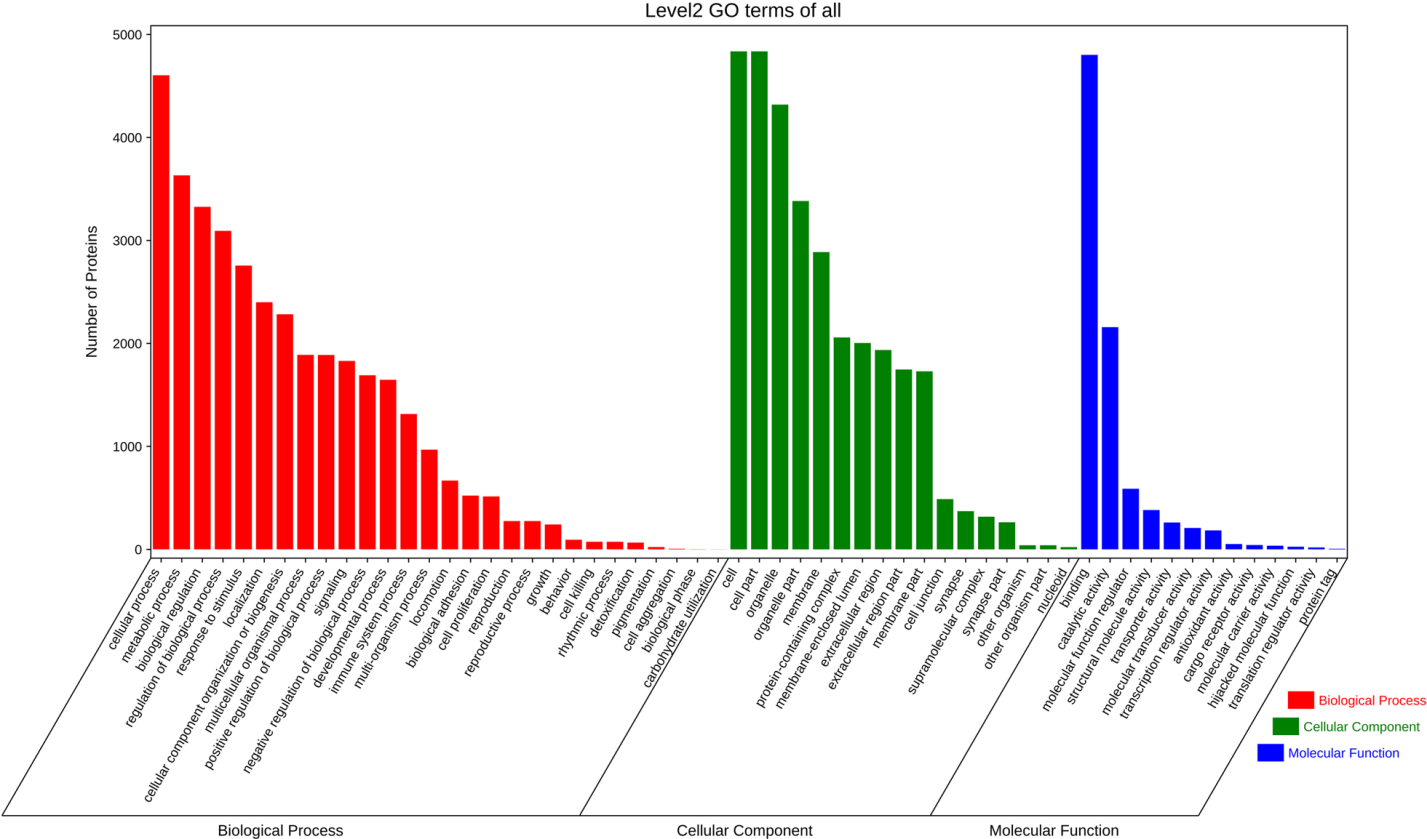
GO function enrichment analysis

GO function enrichment analysis showed the DEPs were mainly involved in biological processes (hidden attributes, catalytic activity, molecular function regulators, structural molecule activity), cellular locations (extracellular regions, organelles, cell membrane), and molecular functions (cell processes, biological regulation, response to stimulus, metabolic processes, regulation of biological processes, immune system responses). The matrix structure components provide tensile strength, extracellular matrix structure components, integrin binding, and protease binding (Fig. 9).

**Fig. 10:**
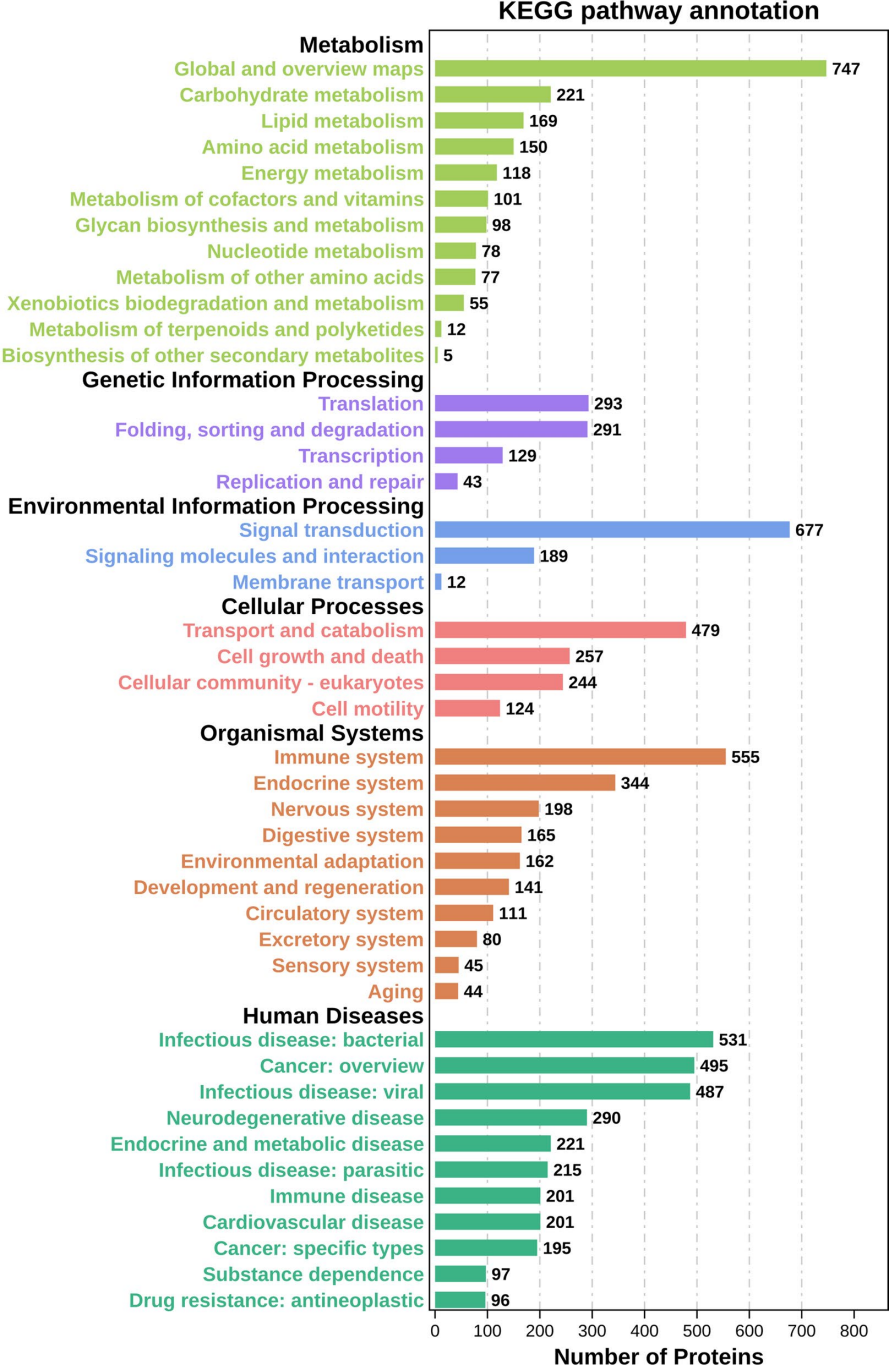
KEGG pathway analysis

KEGG analysis can predict the role of PPI networks in various cell activities. The KEGG PATHWAY database integrates the current knowledge on molecular interaction networks and links the detected molecules with known pathways. KEGG pathway analysis showed DEPs were mainly enriched in starch and sucrose metabolism, ECM–receptor interactions, PI3K-Akt signaling, complement and coagulation cascades, IL-17 signaling, phagosomes, transcriptional misregulation in cancer, and focal adhesions (Fig. 10).

### PPI network analysis

**Fig. 11:**
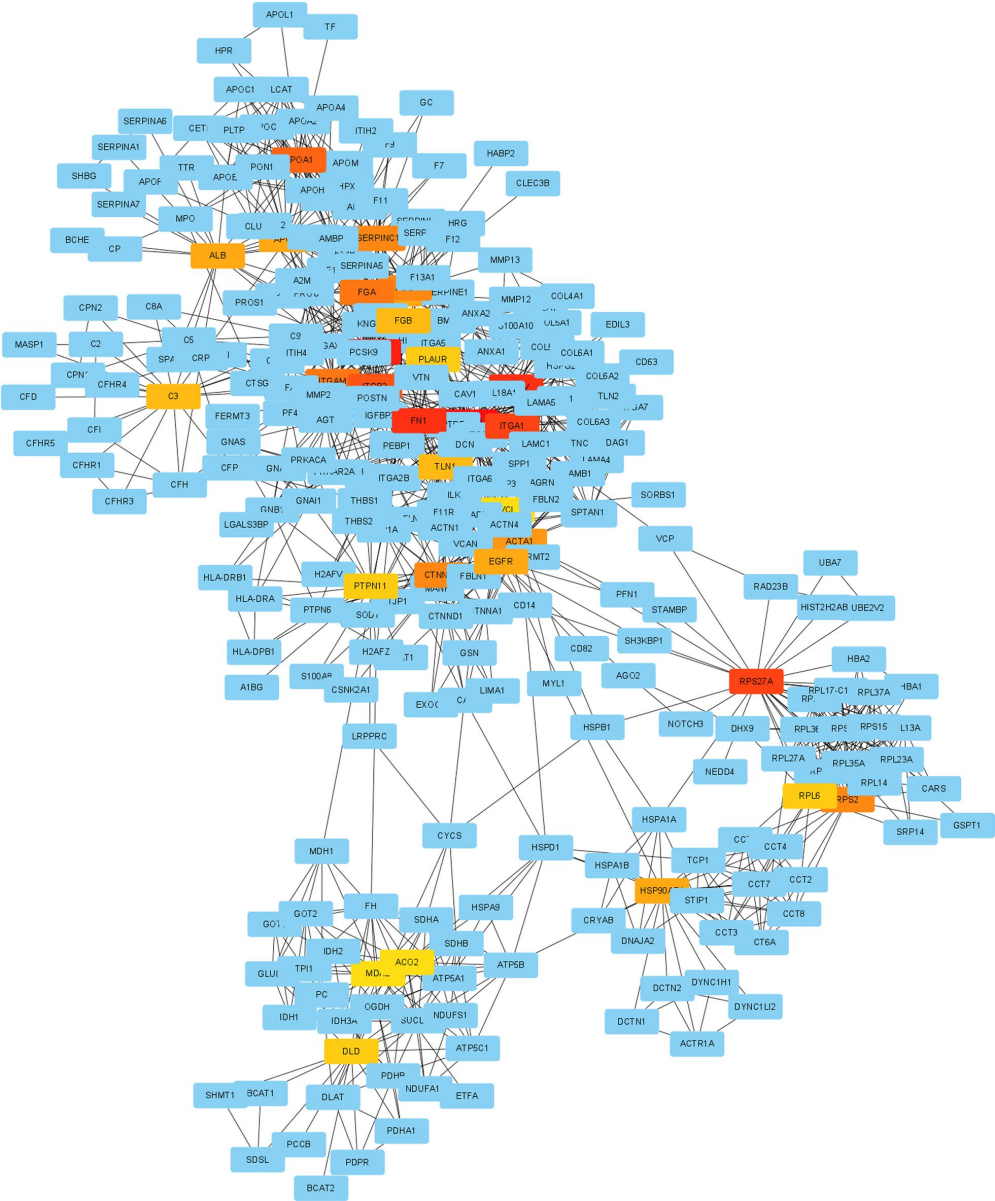
PPI network analysis involves studying PPIs to understand biological processes and functional mechanisms. This network is composed of 109 nodes and 917 edges

Molecular interaction network analysis can reveal the relationships between upregulated or downregulated proteins. The most densely interconnected proteins with the largest number of connections appear at the core of the interaction network and are the focus of the present study. The selected differential genes were mapped to the online STRING, which yielded an interaction network for proteins encoded by differential genes. In this network, nodes represent proteins, while edges or lines indicate PPIs. Such analysis helps reveal the key proteins in the network and how these proteins collaborate in complex biological functions (Fig. 11).

**Fig. 12:**
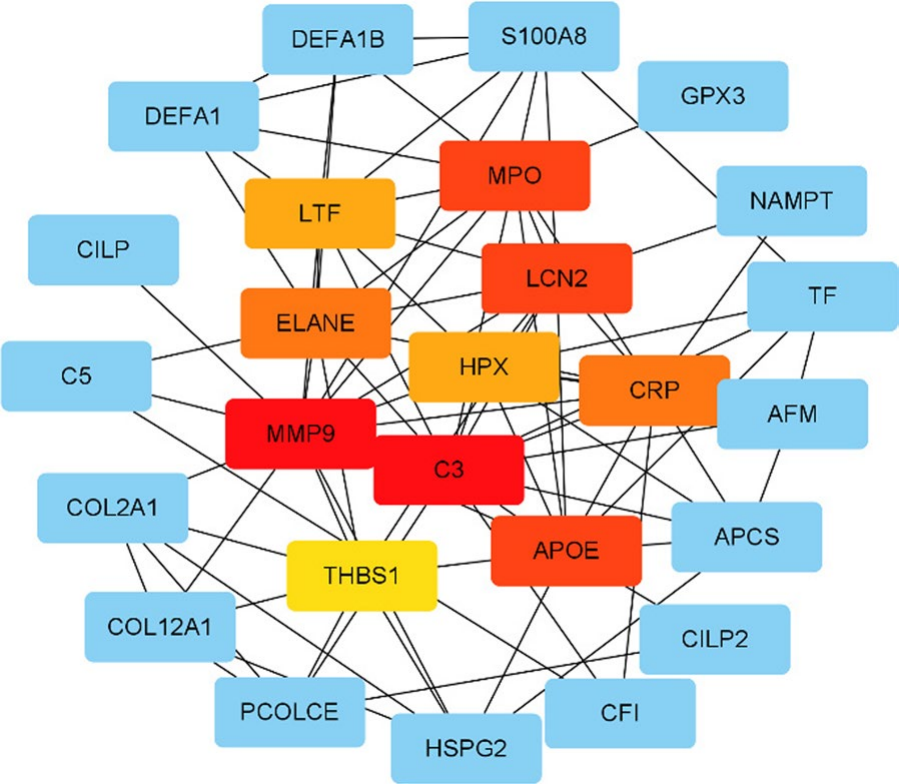
HUB genes screened out by cytoHubba

The cytoHubba plugin MCC (Maximal Clique Centrality) algorithm in Cytoscape was used to calculate the key target genes (Fig. 12) and obtain intersections. The hub genes for necrosis include C3, MMP9, APOE, MPO, LCN2, ELANE, HPX, LTF, THBS1, ITGB1, F2, ITGAV, and APOA1.

### Western blot validation

**Fig. 13:**
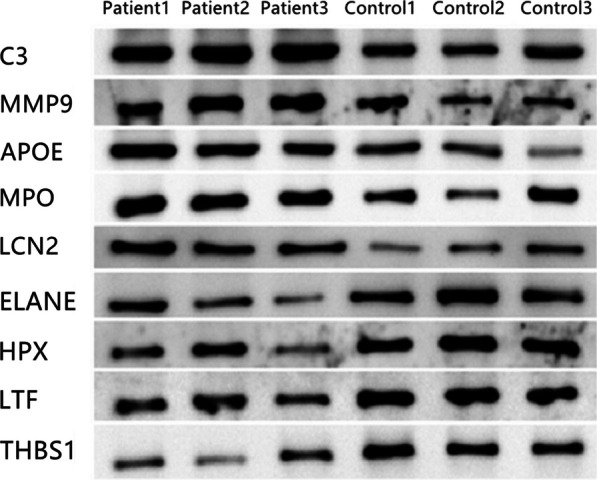
Western blot validation

**Fig. 14:**
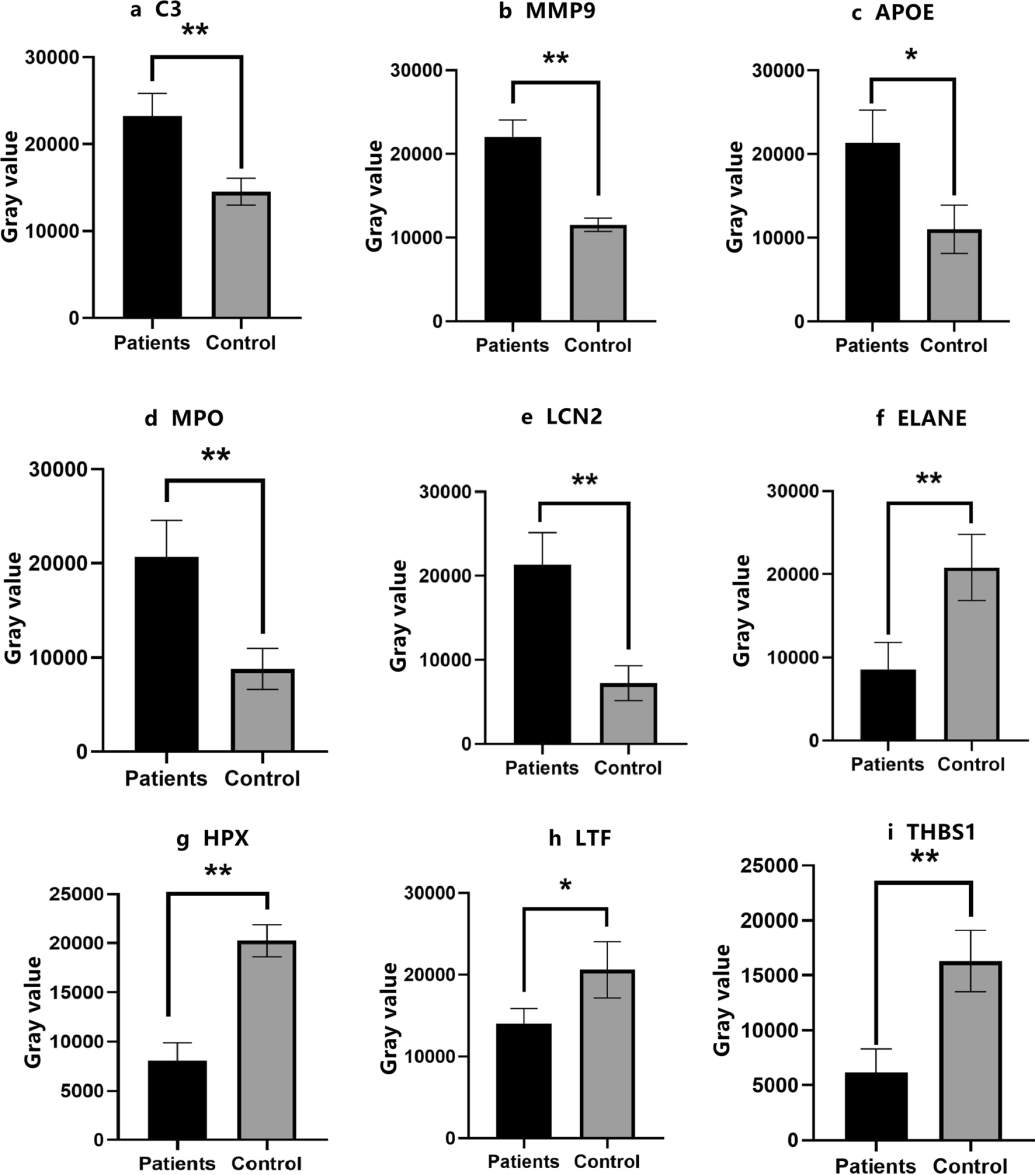
Western blot gray histogram

To validate our proteomic sequencing results, we employed Western blot to determine the expressions of the selected key proteins in ONFH and normal cancellous bone tissue samples [17]. Results showed the expressions of proteins such as C3, MMP9, MPO, LCN2, ELANE, and APOE significantly increased in the ONFH samples. This finding is consistent with our proteomic sequencing results and further supports the potential role of these proteins in the pathogenesis of ONFH that may involve inflammatory responses and changes in the local microenvironment. Furthermore, the expressions of proteins like HPX, LTF, and THBS1 decreased in the ONFH samples (Figs. 13 and 14). Hence, these proteins may play key roles in maintaining bone tissue homeostasis, and their downregulation can further promote the development of ONFH.

## Discussion

ONFH is a complex process regulated by certain key genes, proteins, and signaling pathways. We used DIA quantitative proteomics to comprehensively analyze necrotic femoral head tissues and normal cancellous bone tissues. Our goal was to reveal the group of proteins that play a crucial role in the pathogenesis of ONFH and their potential functions and thus to provide possible targets for future prevention and treatment strategies [18].

We identified 937 significant DEPs between ONFH cartilages and normal cancellous bones, with 422 upregulated and 515 downregulated DEPs. These proteins reflect alterations in various biological processes and molecular functions during ONFH progression. Further bioinformatics analyses revealed potential biological processes and metabolic pathways that may involve these proteins, providing insights into their roles in ONFH. During the data processing, analysis, and validation, we identified 9 key proteins that may be associated with the pathogenesis of ONFH.

GO enrichment analysis with the key proteins helps further understand their roles in biological processes, cellular locations, and molecular functions as well as how they influence the pathogenesis of ONFH by participating in specific biological mechanisms. Certain proteins, such as C3 and MMP9, are enriched in processes such as immune and stimulus responses and play a pivotal role in inflammation and cell damage responses in ONFH [19]. Other proteins like LCN2, ELANE, HPX, and LTF are significantly enriched in extracellular regions, organelles, and cell membranes, which constitute the living environment of many cells in the body and influence cell activity. In terms of molecular functions, proteins such as ITGB1, MPO, and ITGAV are notably enriched in functions like integrin binding and proteinase binding [20]. These functions are crucial for maintaining cell signal transduction and intercellular communication and may play a key role in keeping normal metabolic balance and responding to stimuli.

These findings imply that the upregulated expressions of proteins related to cartilage and the extracellular matrix can be significant drivers in the repair of degenerative cartilage, which is potentially triggered by multiple factors such as hypoxia, ischemia, and metabolic disorder. Additionally, the upregulation of antigen processing genes may indicate a weakened immune response and possibly acts as a protective measure. However, these alterations are insufficient to reverse cartilage degradation. KEGG pathway analysis offers insight into the pathogenesis of ONFH and reveals numerous potential biological processes and signal transduction pathways linked to key proteins [21].

The glycosaminoglycan metabolic pathway is crucial in maintaining cellular energy and repair processes and may be associated with the pathophysiological mechanisms of osteonecrosis [22]. Interactions of extracellular matrix receptors are key pathways to promote cell proliferation and inhibit apoptosis [23]. The PI3K/Akt signaling pathway significantly impacts cellular life activities, including cell proliferation, survival, and differentiation. The activation of complement and coagulation cascades may be involved in immune responses and inflammation processes, especially in osteonecrosis [24]. The IL-17 signaling pathway plays a critical role in regulating inflammatory responses and particularly in promoting the production of inflammatory cytokines and angiogenesis [25]. Focal adhesion, an important structure of the cell skeleton, may be involved in cell growth regulation at the early stages of osteonecrosis [26, 27]. The digestion and absorption of proteins may be related to bone balance disruption and articular cartilage collapse at the late stages of osteonecrosis [28].

We aim to deeply understand the molecular mechanisms of femoral head necrosis. For this goal, we created a PPI network and used online analysis software and network analysis plugins to thoroughly study the interactions and their potential impacts [29].

We identified 13 central genes that may play key roles in femoral head necrosis, including C3, MMP9, and APOE. The positions of these central proteins in the PPI network suggest they may play important roles in multiple biological processes and signaling pathways [30]. For example, C3, a crucial factor in the complement activation pathway, participates in regulating immune responses and assists in pathogen clearance. MMP9, a matrix metalloproteinase, is involved in extracellular matrix degradation and plays a key role in cell migration and tissue remodeling. APOE, a critical lipid metabolism protein, plays a significant role in lipid transport and immune regulation [31]. The discovery of these central genes is critical for us to understand the complex molecular mechanisms of femoral head necrosis. It also provides possible targets for us to formulate prevention and treatment strategies. However, the actual functions of these genes in femoral head necrosis and how to use this information to improve the prevention and treatment of femoral head necrosis still require further research and validation [32, 33]. Our research provides a new knowledge about the molecular mechanisms of femoral head necrosis, but further research is still ongoing. We look forward to future studies that can provide more information about how these genes affect femoral head necrosis, so we can better prevent and treat this disease.

To deepen the validation of the proteomically screened key proteins, we examined their expression differences in the ONFH samples and normal controls via Western blot. These proteins include C3, MMP9, APOE, MPO, LCN2, ELANE, HPX, LTF, and THBS1.

Initial immunofluorescence staining revealed these proteins were broadly distributed across cellular locations such as cytoplasm, nuclei, and cell membranes. Such wide subcellular localization mirrors their potential involvement in various biological processes and their impact on cellular function maintenance. For instance, membrane-bound ITGB1 and ITGAV may be engaged in extracellular interactions, while cytoplasmic MMP9 and MPO can be involved in intracellular signal transduction and metabolic processes. Western blot confirmed the varying expressions of these proteins in ONFH, and some of them (e.g., C3, MMP9, APOE) were upregulated, indicating there were changes in ONFH pathogenesis. Moreover, proteins like LTF and THBS1 were downregulated, which was potentially hinted at impairments in intercellular signaling [34, 35]. These results support our initial proteomics-based findings and enrich our understanding into the roles of these proteins in ONFH. Future research shall explore the precise effects of these proteins on ONFH development and their potential as treatment targets.

### Limitations

Despite some novel and significant findings, it must be acknowledged that our study has certain limitations. Firstly, we only sampled lesion tissues from patients at ARCO stages IIIB-IV, who already required hip joint replacement surgery. The lesion tissues of patients at ARCO stages II-IIIA were not assessed, suggesting our results may predominantly represent the situation of moderate to severe ONFH. Mild- and early-stage femoral head necrosis may involve different molecular mechanisms, which necessitates further research for verification. Secondly, although our original plan was to sample synovial fluid and synovial membrane tissues, we found in practice that synovial fluid was difficult to extract, and the amount was usually insufficient to meet experimental requirements. Additionally, distinguishing between synovium in cases of advanced femoral head necrosis and osteoarthritis was often challenging. As such, we decided to sample load-bearing cartilages instead. These limitations indicate that future research shall be aimed at more comprehensive sampling and analysis, including consideration of ONFH patients at different disease stages, and exploration of more effective methods to obtain sufficient samples of synovial fluid and synovial membrane tissues. This improvement will help us more accurately understand the pathogenesis of ONFH and can guide us to find more effective treatments.

## Conclusions

DIA proteomics and bioinformatics analysis revealed the molecular mechanisms of intra-articular lesions in ONFH. A correlation in the necrotic and load-bearing area cartilages of ONFH at ARCO stages IIIB-IV, and potential key regulatory proteins were identified. These findings help us more deeply understand the pathogenesis of ONFH and may provide important clues for seeking more effective treatment strategies.

## Data Availability

Not applicable.
